# Early constipation predicts faster dementia onset in Parkinson’s disease

**DOI:** 10.1038/s41531-021-00191-w

**Published:** 2021-05-26

**Authors:** M. Camacho, A. D. Macleod, J. Maple-Grødem, J. R. Evans, D. P. Breen, G. Cummins, R. S. Wijeyekoon, J. C. Greenland, G. Alves, O. B. Tysnes, R. A. Lawson, R. A. Barker, C. H. Williams-Gray

**Affiliations:** 1 Department of Clinical Neurosciences, University of Cambridge, Cambridge, UK; 2 Institute of Applied Health Sciences, University of Aberdeen, Aberdeen, UK; 3 The Norwegian Centre for Movement Disorders, Stavanger University Hospital, Stavanger, Norway; 4 Department of Chemistry, Bioscience and Environmental Engineering, University of Stavanger, Stavanger, Norway; 5 Nottingham University Hospital NHS Trust, Nottingham, UK; 6 Centre for Clinical Brain Sciences, University of Edinburgh, Edinburgh, UK; 7 Anne Rowling Regenerative Neurology Clinic, University of Edinburgh, Edinburgh, UK; 8 Usher Institute of Population Health Sciences and Informatics, University of Edinburgh, Edinburgh, UK; 9 Department of Neurology, Haukeland University Hospital, University of Bergen, Bergen, Norway; 10 Translational and Clinical Research Institute, Newcastle University, Newcastle upon Tyne, UK; 11 Wellcome Trust-MRC Cambridge Stem Cell Institute, University of Cambridge, Cambridge, UK

**Keywords:** Risk factors, Parkinson's disease

## Abstract

Constipation is a common but not a universal feature in early PD, suggesting that gut involvement is heterogeneous and may be part of a distinct PD subtype with prognostic implications. We analysed data from the Parkinson’s Incidence Cohorts Collaboration, composed of incident community-based cohorts of PD patients assessed longitudinally over 8 years. Constipation was assessed with the MDS-UPDRS constipation item or a comparable categorical scale. Primary PD outcomes of interest were dementia, postural instability and death. PD patients were stratified according to constipation severity at diagnosis: none (*n* = 313, 67.3%), minor (*n* = 97, 20.9%) and major (*n* = 55, 11.8%). Clinical progression to all three outcomes was more rapid in those with more severe constipation at baseline (Kaplan–Meier survival analysis). Cox regression analysis, adjusting for relevant confounders, confirmed a significant relationship between constipation severity and progression to dementia, but not postural instability or death. Early constipation may predict an accelerated progression of neurodegenerative pathology.

## Introduction

Although Parkinson’s disease (PD) is characterized as a movement disorder, it is associated with significant non-motor features including gastrointestinal (GI) dysfunction^1,2^ such as constipation, dysphagia, sialorrhea, delayed gastric emptying and reflux^3,4^. Constipation is one of the most common non-motor PD symptoms^5^ and can significantly impact on the patient’s quality of life^6^. Individuals with constipation are at greater risk of developing PD^7^ and it is now a recognized feature of prodromal PD^8,9^.

Research efforts on the gut–brain axis have increased dramatically and there is accumulating evidence for a possible role of gut dysfunction in the early pathogenesis of PD^10–12^; however, its link to disease progression has received less attention^13,14^. James Parkinson, in *An Essay on the Shaking Palsy*^15^, reported the amelioration of PD symptoms in two cases following the alleviation of constipation after laxative prescription, which made him question the role of the GI tract in the disease process. More recent studies^16,17^ reporting motor improvement following constipation treatment suggest that, more than 200 years later, the question remains of interest but more basic and clinical research evidence is needed to confirm this association.

It has been proposed that changes in gut function may impact on PD through alteration in the gut microbiota composition^11^. Clinical trials with probiotics (Clinicaltrials.gov identifier NCT04140760) and faecal microbiota transplantation (Clinicaltrials.gov identifier NCT03808389) are being explored as novel therapeutic approaches^18^. The mechanistic basis of this is unclear, but one hypothesis is that early alpha-synuclein pathology in the enteric nervous system in PD leads to reduced gut motility, which results in changes in the microbiome. Both local alpha-synuclein deposition and an altered microbiome may contribute to low-grade gut and systemic inflammation, in turn exacerbating brain inflammation and neurodegeneration and leading to more rapid disease progression^19,20^. While it could be argued that early pathological changes in the enteric nervous system in PD and associated constipation simply reflect a more malignant disseminated disease process, this seems unlikely given that constipation can predate the onset of motor symptoms in PD by 10–16 years^21^.

To date, although there are effective therapies for some of the key motor features of PD, there are still no disease-modifying treatments. At the 10 year longitudinal assessment of an incident population-representative PD cohort (CamPaIGN), only 23% of patients had a good outcome (surviving free of dementia/postural instability), while 68% of patients had developed postural instability and 46% had developed dementia^22^. Constipation is a relatively preventable and treatable aspect of PD, and if found to be causally linked to long-term PD prognosis, warrants further consideration as a target for disease modification.

## Results

Three participants with missing constipation items and two patients with Mini Mental State Examination (MMSE) scores highly suggestive of dementia (MMSE < 18) at the baseline visit were excluded. For survival analysis of postural instability, patients with H&Y3+ at baseline were excluded (*n* = 44). The total sample size was 465 patients for the dementia and mortality analyses and 421 patients for the postural instability analysis. Participants were longitudinally assessed for up to 8.6 years from diagnosis, with an average follow-up time of 5.1 years (SD = 2.5) from diagnosis.

At baseline, 20.9% of patients had ‘minor’ constipation (*n* = 97) and 11.8% of patients had ‘major’ constipation (*n* = 55), while 67.3% of patients (*n* = 313) did not report any constipation (Table 1). Patients with ‘major’ constipation had higher baseline Movement Disorders Society-Unified Parkinson’s Disease Rating Scale (MDS-UPDRS-III) scores compared to the ‘minor’ and no constipation groups (*p* < 0.01). Hoehn and Yahr (H&Y) stage and age were also higher in patients with ‘major’ constipation compared to the no constipation group. Constipated and non-constipated patients did not differ in terms of sex, smoking status, disease duration, time between symptom onset and diagnosis, levodopa equivalent daily dose (LEDD), years of education, MMSE, number of comorbidities at baseline, presence of vascular disease or diabetes, or use of anticholinergic or opiate drugs at baseline.

**Table 1:** Baseline demographic and clinical characteristics of PD patient groups stratified by constipation severity.

	No constipation (*n* = 313)	Minor constipation (*n* = 97)	Major constipation (*n* = 55)	*P* value
Sex (% male)	62.0%	61.9%	54.3%	0.469
Smoking status (% smokers)	5.1%	7.2%	3.6%	0.07
Age at diagnosis	67.2 ± 9.7^♯^ (40.4–96.4)	69.6 ± 9.1 (42.4–85.3)	70.8 ± 8.1^♯^ (51.1–85.4)	0.005^*^
Education (years)	11.8 ± 3.2 (5–22)	12.2 ± 3.5 (7–21)	11.1 ± 2.7 (7–18)	0.150
H&Y stage	1.8 ± 0.7^♯^ (1–5)	1.9 ± 0.6^♯^ (1–4)	2.2 ± 0.8 (1–4)	<0.001^*^
MDS-UPDRS-III	29.4 ± 11.8^₫^ (7–67)	30.9 ± 13.4 (5–68)	36.0 ± 13.7^₫^ (11–74)	0.005^*^
l-Dopa equivalent daily dose (mg)	85.3 ± 139.0 (0–600)	71.1 ± 141.3 (0–700)	94.4 ± 140.1 (0–600)	0.261
Time from diagnosis (years)	0.2 ± 0.3 (0–2.4)	0.2 ± 0.2 (0–1.6)	0.2 ± 0.3 (0–1.7)	0.712
Time from symptom onset to diagnosis (years)	1.7 ± 1.6 (0–18.3)	2.1 ± 2.6 (0.3–22.8)	1.7 ± 1.6 (0–10)	0.066
MMSE	28.4 ± 1.7 (21–30)	28.3 ± 1.8 (21–30)	28.0 ± 1.8 (22–30)	0.087
Number of comorbidities	1.6 ± 1.5 (0–6)	1.4 ± 1.4 (0–5)	1.9 ± 1.7 (0–7)	0.185
Vascular disease	42.8%	44.3%	56.4%	0.175
Diabetes	8.6%	7.2%	7.3%	0.877
Anticholinergic medication	2.9%	1.0%	1.8%	0.560
Opiate medication	3.5%	1.0%	3.6%	0.436

Values shown are mean ± standard deviation and (range). Continuous variables were compared using Kruskal–Wallis test and categorical variables compared using Chi square tests. Significance threshold is *p* < 0.05. Overall statistical differences are represented by * and pairwise comparisons between groups are represented by ^♯^*p* < 0.05, ^₫^*p* < 0.005 and ^∞^*p* < 0.001. *H&Y* Hoehn and Yahr, *MDS-UPDRS-III* Movement Disorders Society-Unified Parkinson’s Disease Rating Scale, *l-Dopa* Levodopa, *MMSE* Mini Mental State Examination.

### Dementia

**Fig. 1:**
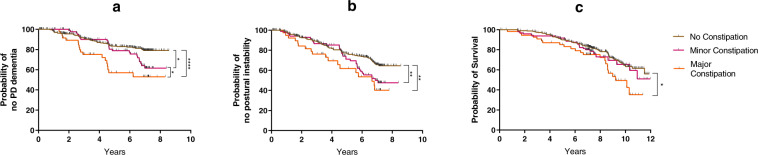
Survival analysis for major outcomes of Parkinson’s disease according to constipation severity. Kaplan–Meier survival analysis for dementia (**a**), postural instability (**b**) and mortality (**c**) in groups stratified by constipation severity. (*, **, **** denote *p* ≤ 0.05, *p* ≤ 0.01, *p* ≤ 0.0001, respectively, log-rank test).

Kaplan–Meier survival analysis indicated faster progression to dementia in more constipated patients (Fig. 1a). At the study endpoint, the cumulative proportion with dementia was 20.9% of patients in the no constipation group versus 38.3% in the ‘minor’ constipation and 47.0% in the ‘major’ constipation group. The no constipation group had the longest mean time to dementia of 7.5 years (95% CI, 7.3–7.8), followed by the ‘minor’ constipation group at 7.1 years (95% CI, 6.6–7.5) and the ‘major’ constipation group with the shortest time of 6.0 years (95% CI, 5.2–6.8). A log-rank test confirmed that survival distributions for the three groups were significantly different (*χ*^2^(2) = 15.364, *p* < 0.0001). Pairwise log-rank comparisons indicated that this difference was most pronounced for the no constipation versus ‘major’ group (‘major’ versus no constipation *χ*^2^(1) = 13.707, *p* < 0.0001; ‘minor’ versus no constipation *χ*^2^(1) = 3.888, *p* = 0.049; ‘minor’ versus ‘major’ constipation *χ*^2^(1) = 3.755, *p* = 0.053). Cox regression analysis confirmed that constipation severity was a significant predictor of dementia outcome, after adjusting for covariates (Table 2; HR = 1.45, *p* = 0.008). Similar significant results were obtained if H&Y stage was included as a covariate instead of MDS-UPDRS-III motor score in the Cox regression model (HR(constipation) = 1.35, *p* = 0.038). To investigate the possibility of selective attrition in the different constipation severity groups, we compared baseline cognitive and baseline motor severity scores between patients who were lost to follow-up without a known dementia outcome (36%) and the group who either reached dementia or remained active at the study’s endpoint (64%). There were no between-group differences in terms of baseline MDS-UPDRS-III and MMSE scores in the ‘minor’ versus ‘major’ constipation groups (data not shown). However in the no constipation group, we observed a marginal difference in baseline MMSE (mean of active group = 28.5, mean of lost to follow-up group = 28.2; *U* = 12,717.5, *Z* = 2.050, *p* = 0.040) and a more significant difference in baseline MDS-UPDRS-III, which was higher in those who were lost to follow-up without a known outcome (mean of active group = 26.9, mean of lost to follow-up group = 33.8; *U* = 7689.0, *Z* = −4.539, *p* < 0.0001). This suggests selective drop-out of patients with more severe motor impairment at baseline in the no constipation group, which could have a confounding effect on our results, but this would not have impacted on the observed difference we found between the ‘minor’ and ‘major’ constipation groups.

**Table 2:** Cox regression models of the predictive role of constipation severity on time to postural instability, dementia and death, adjusting for covariates.

	Dementia (*χ*^2^(6) = 71.912, *p* < 0.0001)			Postural instability (*χ*^2^(5) = 100.414, *p* < 0.0001)			Death (*χ*^2^(6) = 156.136, *p* < 0.0001)		
Variable	HR	95% CI	*P* value	HR	95% CI	*P* value	HR	95% CI	*P* value
Constipation severity	1.45	1.10–1.91	0.008*	1.22	0.95–1.57	0.120	0.93	0.73–1.20	0.568
Age at diagnosis	1.06	1.03–1.09	<0.001*	1.09	1.07–1.12	<0.001*	1.11	1.08–1.14	<0.001*
Sex (female)	0.59	0.37–0.94	0.028*	1.02	0.71–1.47	0.913	0.51	0.35–0.35	0.001*
MDS-UPDRS-III	1.03	1.01–1.04	0.004*	1.03	1.02–1.05	<0.001*	1.04	1.02–1.05	<0.001*
MMSE	0.87	0.79–0.96	0.008*	–	–	–	0.90	0.82–0.98	0.021*
Study centre	1.75	1.12–2.74	0.014*	0.64	0.45–0.91	0.013*	0.89	0.57–1.40	0.618

*HR*, hazard ratio, *CI* confidence interval, *MDS-UPDRS-III* Movement Disorders Society-Unified Parkinson’s Disease Rating Scale, *MMSE* Mini Mental State Examination.

*Significance threshold *p* < 0.05.

### Postural instability

Baseline constipation severity was associated with faster progression to development of postural instability as defined by H&Y3 or higher (Fig. 1b). At the study endpoint, the cumulative percentage of patients with an H&Y score ≥3 was 35.4% in the no constipation group versus 52.3% in the ‘minor’ constipation and 59.8% in the ‘major’ constipation group. The no constipation group had the longest mean time to H&Y3+ of 6.9 years (95% CI, 6.6–7.3) compared to 6.4 years (95% CI, 5.9–7.0) in the ‘minor’ constipation group and 5.5. years (95% CI, 4.7–6.3) in the ‘major’ constipation group. H&Y3+ survival distributions for the three constipation severity groups were significantly different (*χ*^2^(2) = 9.882, *p* = 0.007). Pairwise log-rank comparisons indicated that this was mainly driven by the difference between the ‘major’ versus the no constipation group (‘major’ versus no constipation *χ*^2^(1) = 7.294, *p* = 0.007; ‘minor’ versus no constipation *χ*^2^(1) = 4.314, *p* = 0.038; ‘minor’ versus ‘major’ constipation *χ*^2^(1) = 1.310, *p* = 0.252). However, Cox regression analysis did not confirm a statistically significant association between constipation severity and time to postural instability when adjusting for age, sex, baseline MDS-UPDRS-III and cohort study (Table 2).

### Death

The association between constipation and time to death was also investigated (Fig. 1c). At the study endpoint, the cumulative mortality proportions were 44.1% in the no constipation group, 49.1% in the ‘minor’ constipation and 64.7% in the ‘major’ constipation group.

Mean time to death was 10.0 years (95% CI, 9.7–10.4) in the no constipation group, and 9.7 years (95% CI, 9.0–10.5) in the ‘minor’ constipation group, while the ‘major’ constipation group had the shortest mean time to death of 8.7 years (95% CI, 7.8–9.6). The survival distributions for the three constipation severity groups were significantly different (*χ*^2^(2) = 6.286, *p* = 0.046), which was driven by the difference between the ‘major’ versus the no constipation group (*χ*^2^(1) = 6.105, *p* = 0.013), with no differences between the ‘minor’ versus no constipation group and between the ‘minor’ and ‘major’ groups (*χ*^2^(1) = 0.537, *p* = 0.463; *χ*^2^(1)=1.922, *p* = 0.166, respectively). This association did not withstand Cox regression analysis after correction for the potentially confounding effects of age, sex, MDS-UPDRS-III, MMSE and study centre (Table 2).

### Alternate measures of autonomic and GI dysfunction

Because early autonomic dysfunction has been associated with more rapid disease progression and shorter survival in patients with PD^23^, we investigated whether constipation was a proxy measure of autonomic dysfunction in our sample. We first conducted a chi square test of independence between constipation severity and postural hypotension. Because 22.2% of expected cell frequencies were lower than five in both tests, we dichotomized the variables and repeated the analysis. Postural hypotension was not significantly higher (*p* = 0.243) in the constipated group (23.1%) compared with the non-constipated group (17.0%). Cox regression models for groups stratified by postural hypotension (PICNICS cohort, *n* = 278) did not show any association with time to dementia (HR = 0.58, *p* = 0.238), postural instability (HR = 1.30, *p* = 0.305) or death (HR = 0.92, *p* = 0.674). We also analysed dysphagia as an alternative symptom of GI dysfunction associated with more advanced disease. Dysphagia was significantly higher (*p* = 0.031) in the constipated group (22.4%) compared with the non-constipated group (14.4%). However, Cox regressions indicated no relationship between dysphagia and time to dementia (HR = 1.03, *p* = 0.884), time to postural instability (HR = 1.01, *p* = 0.942) or death (HR = 1.17, *p* = 0.353).

## Discussion

We investigated the relationship between early constipation and subsequent disease progression in a representative population of incident, community-based PD cases and demonstrated that constipation severity at disease onset predicts faster progression to dementia. Our data also suggest a similar trend for an association with faster motor progression to H&Y stage 3 and death, although these associations did not reach statistical significance in Cox regression modelling with correction for covariates. Our findings support previous research on the association of constipation with disease duration/severity^24,25^, but our study investigates the predictive relationship of constipation severity at the time of diagnosis with the key disease milestones of dementia, postural instability and death. To date, only one other study by Jones et al.^14^ has analysed the longitudinal relationship between severity of GI symptoms and cognitive impairment in newly diagnosed PD patients. Jones et al. used a composite GI symptom score rather than constipation per se and explored the relationship between longitudinal GI scores and cognitive performance over time, rather than assessing whether baseline GI symptoms predict subsequent cognitive outcome. Our results are in line with their study which reported that a higher frequency of GI symptoms was associated with worse cognitive performance over 5 years as well as mild cognitive impairment and PD dementia (PDD). Similar to our results, their findings suggested that cognitive function was not significantly related to non-GI autonomic symptoms, indicating that these observations are specific to the GI system rather than to autonomic dysfunction per se.

The pathophysiological basis of this association between early gut dysfunction and PD progression is unknown but accumulation of alpha-synuclein protein aggregates in the gut in early stages of PD has led to the hypothesis that, in a subset of patients, PD pathology may begin in the gut before spreading to connected areas of the nervous system^26–28^. Colonic alpha-synuclein is associated with chronic constipation and pathophysiological changes in the intestinal wall^20,29^. Patients with early PD have been shown to have increased intestinal permeability, altered microbiota composition, increased bacterial translocation through the gut wall, and higher levels of inflammatory cytokines in the GI tract^20^. The presence of lipopolysaccharides (LPS) and related bacterial proteins has been shown to be increased in brains of patients with Alzheimer’s Disease, indicating that endotoxins can access the brain^30^. Furthermore, peripheral administration of LPS in humans can cause rapid microglial activation and increase in peripheral inflammatory cytokines^31^. So, it is possible that gut changes in PD may trigger a systemic chronic immune response which, in turn, promotes faster progression^12,32,33^. While the association between the microbiome and dementia risk in PD has not yet been investigated in long-term studies, cognitive dysfunction (particularly impaired attention, mental flexibility and executive function) has been reported to be associated with gut microbiota composition in cross-sectional and longitudinal studies in non-PD cohorts (mainly composed of obese individuals)^34–37^. In a population-based cohort study of patients with inflammatory bowel disease, patients had a higher risk of subsequent development of dementia, and with a younger average age of onset than the general population^38^. In PD, Wijeyekoon et al.^39^ have found elevated bacterial endotoxin levels in the serum of PD patients compared to controls, particularly in those patients at higher risk of an early dementia. Hence early constipation and associated translocation of bacterial products into the circulation may have particular relevance for cognitive decline in PD.

An alternative explanation for our observation is that the early constipation associated with a more rapid development to a dementia reflects a more malignant disseminated form of PD, with widespread evolving alpha-synuclein pathology from disease onset. This fits with the observation that constipation has been associated cross-sectionally with disease severity at baseline^40^ but arguing against this interpretation is the fact that in our study, constipation was associated with dementia independently of disease motor severity at baseline. Of note, while constipated patients had significantly higher rates of dysphagia (an alternate measure of GID), at baseline, this was not independently associated with time to dementia, postural instability or death. Similarly, baseline postural hypotension (an alternative measure of autonomic dysfunction) was not associated with later disease outcomes. Importantly, our data indicated no difference between constipation groups in terms of disease duration, or motor symptom onset to diagnosis time, suggesting that those with ‘major’ constipation were not temporally more advanced in their disease course. Furthermore, constipation commonly occurs as a prodromal symptom many years before the onset of the typical motor and non-motor features of PD^21^, which suggests it is not merely an indicator of more advanced disease or dysautonomia.

Increasing evidence of distinct subtypes of PD with differential progression trajectories holds important clinical implications^41,42^. Fereshtehnejad et al.^43^ postulated that PD presenting features can be clustered into three PD subtypes with different prognostic trajectories, mainly motor/slow progression (cluster I), diffuse/malignant (cluster II) and intermediate (cluster III). Despite equivalent age and disease duration between groups, patients in the malignant and intermediate clusters presented with high constipation scores and other non-motor symptoms such as orthostatic hypotension and REM sleep behaviour disorder (RBD), unlike the slow progression subtype^44^. Furthermore, clusters II and III were associated with faster cognitive decline. These results support our finding that minor and major constipation problems at diagnosis are associated with faster onset to PDD.

Major strengths of this study are the use of a large population-representative cohort with incident, community-based PD and a prospective follow-up period of up to 8 years after diagnosis. This minimized selection bias and increased diagnostic certainty and information on long-term prognosis. However, the study has limitations. Despite the prolonged longitudinal follow-up, non-survival distributions did not reach 50% of our sample and extended follow-up would allow for an increased number of events which will then permit calculations of median times to outcome. Attrition is a common issue in longitudinal studies and can introduce bias if there is selective drop out of cases at higher risk of the outcome of interest. We did find that among cases with no constipation, those who were lost to follow-up without a dementia outcome had more motor impairment at baseline. It is possible that this led to an underestimation of cumulative dementia incidence in the no constipation group. GI comorbidities and laxative use may have influenced constipation severity in these cohorts, but detailed information on these factors was not available. However, we investigated the potential confounding effects of vascular disease, diabetes, anticholinergic and opiate medication that have previously been associated with cognitive decline or constipation severity and found no difference between the constipation groups. We also did not have data on diet, which may affect bowel function, the microbiome and risk of cognitive impairment independently. However, irrespective of the aetiology of constipation, and the effects of GI treatment on this, we confirmed a link with faster subsequent disease progression, which supports the hypothesis that measures to reduce constipation may be beneficial in terms of disease modification, assuming these are causally linked.

Given the evidence that RBD is an important prodromal and prognostic feature in PD and seems to be associated with constipation^42,45^ it would have been interesting to have data on RBD status. Unfortunately, polysomnography studies are difficult to incorporate in large longitudinal studies and the REM Sleep Behavior Disorder Screening Questionnaire (RBDSQ)^46^ was only published after the start of these cohort studies; thus, these data were not collected. A final limitation is the lack of a dedicated constipation/GI questionnaire for stratification of the sample. We used the constipation item of the MDS-UPDRS, which is commonly used in PD research studies^24^. A number of different methods for evaluating constipation have been used by previous authors, such as the SCOPA-AUT^14^ or the NMSS^47^. To date, no PD-specific psychometric instrument that reflects ROME-IV^48^ criteria is available. Objective measures of gut transit time may be more sensitive for detecting colonic dysfunction than symptom-based questionnaires^4^, but are difficult to incorporate in large longitudinal studies. It is possible that our method of quantifying constipation led to an underestimation of incidence and severity, and further studies with more detailed constipation assessment are needed to validate our findings.

In summary, our findings support the importance of studying constipation in PD, showing that the presence and severity of this symptom around the time of diagnosis is predictive of subsequent progression to dementia. This supports the hypothesis that, in a subset of patients, early constipation may be associated with an accelerated progression of neurodegenerative pathology in the brain, although the mechanism by which this occurs is currently unknown.

## Methods

We used data from the Parkinson’s Incidence Cohorts Collaboration (PICC), a project that has pooled data from six prospective PD incidence cohorts in Northern Europe (CamPaIGN, ICICLE-PD, NYPUM, ParkWest, PICNICS and PINE)^49^. All patients were diagnosed with idiopathic PD using UK Parkinson’s Disease Society Brain Bank criteria. Each study collected demographic, clinical and genetic data close to the time of diagnosis and at regular follow-up visits thereafter. Ethical approval for each study was obtained from the relevant local ethics committees and all participants gave written informed consent. Data on constipation at baseline were available for two datasets: PICNICS (*n* = 280) and ParkWest (*n* = 190). PICNICS follow-up assessments were performed every 18 months up to five visits, and ParkWest follow-up assessments were performed every 12 months up to seven visits. Motor features were assessed using the MDS-UPDRS-III in the PICNICS cohort and the UPDRS-III in the ParkWest cohort (the latter was converted to MDS-UPDRS-III score using the formula proposed by Goetz et al.)^50^. The MMSE was used in both cohorts to assess global cognitive function. The number of comorbidities at baseline was quantified in terms of the number of organ systems affected using the Cumulative Illness Rating Scale (CIRS)^51^ for PICNICS and the Charlson Comorbidity Index^52^ (CCI) for ParkWest. Presence of vascular disease and diabetes at baseline was also specifically explored given that they have been implicated in dementia risk. Vascular conditions included hypertension, angina, myocardial infarction, peripheral vascular disease, cerebrovascular disease (transient ischaemic attack, stroke) and atrial fibrillation/cardiac arrhythmia. LEDD was calculated according to an adapted version of the Tomlinson formula^53^ for both cohorts. Use of anticholinergic drugs and opiate-based drugs was recorded at baseline. Constipation presence and severity was assessed using item 1.11 of the MDS-UPDRS in the PICNICS study and a comparable interview-based question for ParkWest with a severity scale of 0–3. According to scores on this constipation item at the participants’ baseline visit, the sample was stratified into three groups: no constipation (score = 0), minor (score = 1) and major (score ≥ 2) constipation. Dysphagia at baseline was considered as an alternative GI-based predictor of outcome and was assessed using the MDS-UPDRS item 2.3 for PICNICS and a comparable question in ParkWest with a 0–3 severity scale. Postural hypotension was considered as an alternative autonomic predictor of outcome and was assessed using the MDS-UPDRS item 1.12 for the PICNICS cohort but was not assessed in ParkWest. Patients were stratified as ‘none’, ‘minor’ and ‘major’ for dysphagia and postural instability using the same methodology as for constipation.

Outcomes for this study were the development of PDD, postural instability or death. Postural instability was defined as a consistent score of 3 or higher on the H&Y scale, with date of postural instability calculated as the midpoint between the visit at which it was reached, and the preceding visit. PDD onset was defined using the MDS PDD criteria^54^, with the date of dementia determined similarly using the midpoint rule. Where patients were lost to follow-up from the study, dementia diagnoses were determined from clinical records or death certificates (*n* = 16/48 PICNICS; *n* = 2/44 ParkWest), with date of dementia diagnosis being determined using the midpoint between the date that dementia was first recorded and the preceding clinical visit or research appointment, as appropriate. Mortality status was obtained by continual follow-up, including review of clinical notes and death certificates. Time to outcome (in years) was computed by calculating the difference in days between outcome date and PD diagnosis date and dividing by 365.

### Statistical analysis

Data were analysed using IBM SPSS Statistics (Version 25). A significance threshold of *p* < 0.05 was used. Graphs were generated using Graphpad Prism version 8. Because Shapiro–Wilk’s test did not confirm distribution normality for continuous variables we used the Kruskal–Wallis test for between-group comparisons. Chi square tests of independence were used for between-group comparisons for sex, smoking status, dysphagia, postural hypotension, presence of vascular disease and diabetes, use of opiate or anticholinergic medication.

Kaplan–Meier survival analyses of PD outcomes were performed in groups stratified according to constipation severity, with date of diagnosis as time = 0, and censoring at the last assessment for dementia and postural instability analyses and at the study endpoint for the mortality analysis. The proportional hazards assumption was checked by visual inspection of Kaplan–Meier plots by level of covariates. Survival to the events did not exceed 50% at the longest time point in the no constipation and ‘minor’ constipation groups; thus, median times could not be calculated and mean times are reported instead. Pairwise log-rank comparisons were conducted to compare Kaplan–Meier curves between groups. Cox regression analyses were performed to control for relevant covariates (age, sex, study cohort, baseline motor severity (MDS-UPDRS-III) and baseline global cognitive score (MMSE). Constipation was used as an interval dependent variable. H&Y score was not included in the model because of collinearity with MDS-UPDRS-III motor score. We tested an interaction effect between study and constipation and it was non-significant, thus not included in the regression models. For the dementia analysis, education was also added to the regression model but was found to be not significant and thus not included.

### Reporting summary

Further information on research design is available in the Nature Research Reporting Summary linked to this article.

## Supplementary information

Reporting Summary

## Data Availability

Anonymized data for all six cohorts included in PICC are available upon reasonable request by any qualified investigator.

## Code availability

No custom codes were used. All software and packages, their versions, relevant specification and parameters are stated in the ‘Methods' section.
