# Supplementary material for: Early constipation predicts faster dementia onset in Parkinson’s disease
Source: NPJ Parkinsons Dis. 2021 May 26;7:45. doi: 10.1038/s41531-021-00191-w (PMC8154963; doi:10.1038/s41531-021-00191-w)
Supplement: Supplementary file 1 — Reporting Summary [file 41531_2021_191_MOESM1_ESM.pdf]

nature research

Corresponding author(s): Marta Camacho

Reporting Summary

Nature Research wishes to improve the reproducibility of the work that we publish. This form provides structure for consistency and transparency in reporting. For further information on Nature Research policies, see Authors & Referees and the Editorial Policy Checklist.

Statistical parameters

When statistical analyses are reported, confirm that the following items are present in the relevant location (e.g. figure legend, table legend, main text, or Methods section).

n/a	Confirmed	
☐	☒	The exact sample size (*n*) for each experimental group/condition, given as a discrete number and unit of measurement
☐	☒	An indication of whether measurements were taken from distinct samples or whether the same sample was measured repeatedly
☐	☒	The statistical test(s) used AND whether they are one- or two-sided<br>*Only common tests should be described solely by name; describe more complex techniques in the Methods section.*
☐	☒	A description of all covariates tested
☐	☒	A description of any assumptions or corrections, such as tests of normality and adjustment for multiple comparisons
☐	☒	A full description of the statistics including central tendency (e.g. means) or other basic estimates (e.g. regression coefficient) AND variation (e.g. standard deviation) or associated estimates of uncertainty (e.g. confidence intervals)
☐	☒	For null hypothesis testing, the test statistic (e.g. *F*, *t*, *r*) with confidence intervals, effect sizes, degrees of freedom and *P* value noted<br>*Give P values as exact values whenever suitable.*
☒	☐	For Bayesian analysis, information on the choice of priors and Markov chain Monte Carlo settings
☒	☐	For hierarchical and complex designs, identification of the appropriate level for tests and full reporting of outcomes
☐	☒	Estimates of effect sizes (e.g. Cohen's *d*, Pearson's *r*), indicating how they were calculated
☐	☒	Clearly defined error bars<br>*State explicitly what error bars represent (e.g. SD, SE, CI)*

Our web collection on statistics for biologists may be useful.

Software and code

Policy information about availability of computer code

Data collection	NA
Data analysis	Data was analysed using IBM SPSS Statistics (Version 25). Graphs were generated using Graphpad Prism version 8.

For manuscripts utilizing custom algorithms or software that are central to the research but not yet described in published literature, software must be made available to editors/reviewers upon request. We strongly encourage code deposition in a community repository (e.g. GitHub). See the Nature Research guidelines for submitting code & software for further information.

Data

Policy information about availability of data

All manuscripts must include a data availability statement. This statement should provide the following information, where applicable:

- Accession codes, unique identifiers, or web links for publicly available datasets
- A list of figures that have associated raw data
- A description of any restrictions on data availability

Anonymized data for all six cohorts included in PICC is available upon reasonable request by any qualified investigator.

Field-specific reporting

Please select the best fit for your research. If you are not sure, read the appropriate sections before making your selection.

☒ Life sciences ☐ Behavioural & social sciences

For a reference copy of the document with all sections, see nature.com/authors/policies/ReportingSummary-flat.pdf

Life sciences

Study design

All studies must disclose on these points even when the disclosure is negative.

Sample size

This is an observational study that analysed data from a sample of population-representative individuals with Parkinson's Disease (PD). We used data from the Parkinson's Incidence Cohorts Collaboration (PICC), a project which has pooled data from six prospective PD incidence cohorts in Northern Europe (CamPaIGN, ICICLE-PD, NYPUM, ParkWest, PICNICS, and PINE). Data on constipation at baseline was available for two datasets: PICNICS (n=280) and ParkWest (n=190). PICNICS follow-up assessments were performed every 18 months up to 5 visits, and ParkWest follow-up assessments were performed every 12 months up to 7 visits. Participants were longitudinally assessed for up to 8.6 years from diagnosis, with an average follow-up time of 5.1 years (SD=2.5) from diagnosis.

Data exclusions

All patients were diagnosed with idiopathic PD using UK Parkinson's Disease Society Brain Bank criteria. Data on constipation at baseline was available for two datasets: PICNICS and ParkWest. Three participants with missing constipation items and two patients with MMSE scores highly suggestive of dementia (MMSE<18) at the baseline visit were excluded. For survival analysis of postural instability, patients with H&Y3+ at baseline were excluded (n=44). The total sample size was 465 patients for the dementia and mortality analyses and 421 patients for the postural instability analysis.

Replication

We analysed pooled data from a data consortium (Parkinson's Incidence Cohorts Collaboration - PICC) to increase sample size. We did not perform replication studies.

Randomization

Not applicable, this is an observational study of patients with Parkinson's disease, with longitudinal assessments.

Blinding

Not applicable, this is an observational study of patients with Parkinson's disease, with longitudinal assessments.

Materials & experimental systems

Policy information about availability of materials

n/a	Involved in the study
☒	☐ Unique materials
☒	☐ Antibodies
☒	☐ Eukaryotic cell lines
☒	☐ Research animals
☐	☒ Human research participants

Human research participants

Policy information about studies involving human research participants

Population characteristics

In this study, we reported the following population characteristics: Sex, Smoking Status, Age at PD diagnosis, Education (years), H&Y stage, MDS-UPDRS-III, L-Dopa Equivalent Daily Dose (mg), Time from diagnosis (years), Time from symptom onset to diagnosis (years), MMSE, Number of comorbidities, Vascular Disease, Diabetes, Anticholinergic medication, Opiate medication. Of these, the following were identified as relevant co-variates: age, sex, study cohort, baseline motor severity (MDS-UPDRS III) and baseline global cognitive score (MMSE). These covariates were included in the Cox regression models.

Method-specific reporting

n/a	Involved in the study
☒	☐ ChIP-seq
☒	☐ Flow cytometry
☒	☐ Magnetic resonance imaging
